# Supplementary material for: Selective extinction against redundant species buffers functional diversity
Source: Proc Biol Sci. 2020 Jul 22;287(1931):20201162. doi: 10.1098/rspb.2020.1162 (PMC7423665; doi:10.1098/rspb.2020.1162)
Supplement: Supplementary Information [file rspb20201162supp2.docx]

**Supplementary Information**

**Selective extinction against redundant species buffers functional diversity**

Catalina Pimiento^1,2^*, Christine D. Bacon^3,4 a^ *, Daniele Silvestro^3,4,5^, Austin Hendy^6^, Carlos Jaramillo^2,7,8^, Alexander Zizka^4,9^, Xavier Meyer^5,10^, Alexandre Antonelli^3,4,11^

^1^ Department of Biosciences, Swansea University, Swansea, SA2 8PP, UK.

^2^ Smithsonian Tropical Research Institute, Box 0843-03092, Balboa, Ancon, Republic of Panama.

^3^ Department of Biological and Environmental Sciences, University of Gothenburg, Box 461, SE-405 30 Gothenburg, Sweden.

^4^ Gothenburg Global Biodiversity Centre, Box 461, SE-405 30 Gothenburg, Sweden.

^5^ Department of Biology, University of Fribourg, Fribourg, Switzerland.

^6^ Natural History Museum of Los Angeles County, Los Angeles, CA, 90007, USA.

^7^ Equipe de Paléontologie, Institut des Sciences de l’Évolution de Montpellier, University of Montpellier, Place Eugène Bataillon, 34095 Montpellier Cedex 05, France.

^8^ Institut des Sciences de l’Évolution de Montpellier, University of Montpellier, Place Eugène Bataillon, 34095 Montpellier Cedex 05, France.

^9^ German Center for Integrative Biodiversity Research (iDiv) Halle Jena Leipzig, 04103, Leipzig, Germany.

^10^ Department of Integrative Biology, University of California, Berkeley, CA 94720, USA.

^11^ Royal Botanical Gardens Kew, TW9 3AE Richmond, United Kingdom.

*Contributed equally to this work.

Author for correspondence: Christine D. Bacon, christinedbacon@gmail.com.

**Content:**

Supplementary methods p. 2

Figure S1 p. 7

Figure S2 p. 8

Figure S3 p. 9

Figure S4 p. 10

Figure S5 p. 11

Figure S6 p. 12

Figure S7 p. 13

Figure S8 p. 14

Table S1 p. 15

Table S2 p. 16

Table S3 p. 17

Supplementary references p. 18

Dataset p. 18

**Supplementary Methods**

***Preservation and rate shifts.*** Preservation was modeled as a Poisson process with estimated preservation rates expressing the expected number of fossil occurrences per sampled lineage per time unit (here 1 Myr). We used a time-variable preservation process where independent preservation rates were estimated in each epoch. The estimated preservation rates were well within the range of values used in simulation studies and yielded a strong performance of the PyRate method [62] (figure S5), thus we consider the result robust to sampling biases. We used a reversible-jump MCMC (RJMCMC) algorithm, as implemented in PyRate to infer the number and temporal placement of rate shifts in the birth-death model, which allowed us to estimate significant changes in the speciation and extinction rates through time. The number of shifts and their temporal placement were jointly estimated in the analyses and their statistical significance is supported by log Bayes factors greater than six. In these analyses rate shifts are estimated in continuous time rather than within predefined time bins. We ran 25 million RJMCMC iterations, sampling every 1,000 iterations. We summarized the results by plotting the marginal rates through time, calculated within arbitrarily small time bins set to 0.1 Myr.

***Sensitivity analyses for functional diversity.*** It has been stated in the literature that the number and type of traits used can profoundly affect functional diversity measures [69, 70]. Our trait selection was based on the ecological roles of mollusks in ecosystems (table S1). Nevertheless, to test the effect of our trait selection on our results, we repeated our functional redundancy analyses by iteratively removing one and two traits at a time. Altering the number of traits did not change the pattern observed in functional redundancy (figure S7).

***Comparative simulations for functional diversity.*** Functional diversity indices (i.e., number of FEs, functional redundancy, over-redundancy and functional richness) per time bin were compared with null expectations to test whether observed functional changes over time were different than expected. To do so, we ran a set of simulations in which we randomized the identity of species 1,000 times, while leaving the diversity pattern through time unchanged. We then repeated all functional diversity analyses using these randomized sets of data (figure S3). Finally, we compared the observed data and the simulated data by counting the fraction of simulations in which each functional diversity metric from simulated datasets was greater that the observed one (table S2). We considered the observed metric significantly greater than expected when it was greater than 97.5% of the simulated datasets.

***Preservation and abundance tests***. We tested the effect of preservation on trait-frequency by plotting the number of occurrences per species (a proxy of preservation potential) against trait states. We found limited differences across traits or states (figure S6). Therefore, we determine that preservation does not to have a major effect on our results.

***Sensitivity of the MTE results to imbalanced species occupancy***. We ran a sensitivity test to assess whether the imbalance between rare and abundant species had an impact on our estimated correlation between redundancy and extinction rates (MTE model). First, we calculated occupancy as the number of grid cells (1x1 degree) in which fossils of each species were found. Second, we categorized species occupancy in three classes: 1) species occupying one cell [low occupancy]; 2) between 2 and 5 cells [medium occupancy]; and 3) more than 5 cells [high occupancy]. As predicted, there was a strong imbalance between occupancy classes, with 2425, 1637, and 358 species in each occupancy class, respectively. Third, to assess whether this imbalance affected the results of the MTE analyses, we generated 10 additional datasets in which species in the low and intermediate occupancy categories were subsampled to reach the number of high occupancy species (358 species; the lowest number of species in all categories). Finally, we analyzed the subsampled datasets under the MTE model to verify if the effect of the three traits found to correlate significantly with extinction based on the full dataset (i.e., redundancy, body size, and environment) were a result of an over-representation of rare species. We therefore ran analyses including only these three traits and turning off the variable selection (i.e. setting P(*I* = 1) = 1) and combined the results across the 10 replicates. The resulting estimated effects of redundancy, body size, and environment remained highly consistent with those inferred from the full set of mollusk species (Figure S8). Based on this analysis, we conclude that our MTE results are robust, and that differences in occupancy did not affect the effect that redundancy and traits had on extinction.


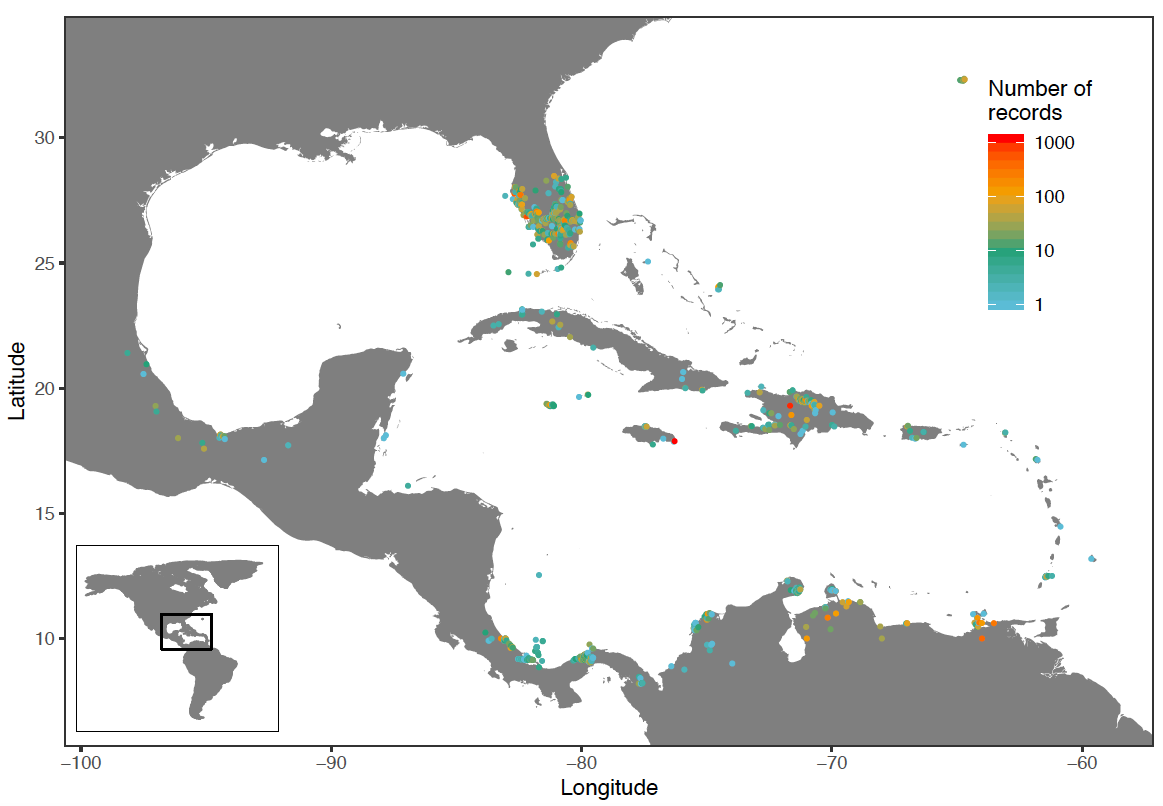


**Figure S1. The study area and number of records per site.** The study area was delimited using the marine ecoregions of the world (Spaulding et al. 2007) and spans between 5.7°N and 35.7°S. Due to fluctuating sea levels during the period examined, many occurrences fall within currently emerged land. Warmer colors show a larger number of records per site and recent occurrence records are extant collections.

**Figure. S2. Timing and statistical significance of origination and extinction through time.** The PyRate analysis estimated (within the Neogene and Quaternary) multiple shifts in speciation and extinction rates. The timing and statistical significance of the rate shifts is inferred from the sampling frequency of rate shifts in the posterior samples. The dashed lines indicate the threshold levels corresponding to a log Bayes factor = 2 (i.e. positive support for a rate shift; bottom line) and log BF = 6 (strong support, upper line). The posterior distributions of the times of rate shifts indicate that there is a high level of confidence in these estimates, even as they incorporate dating uncertainties.


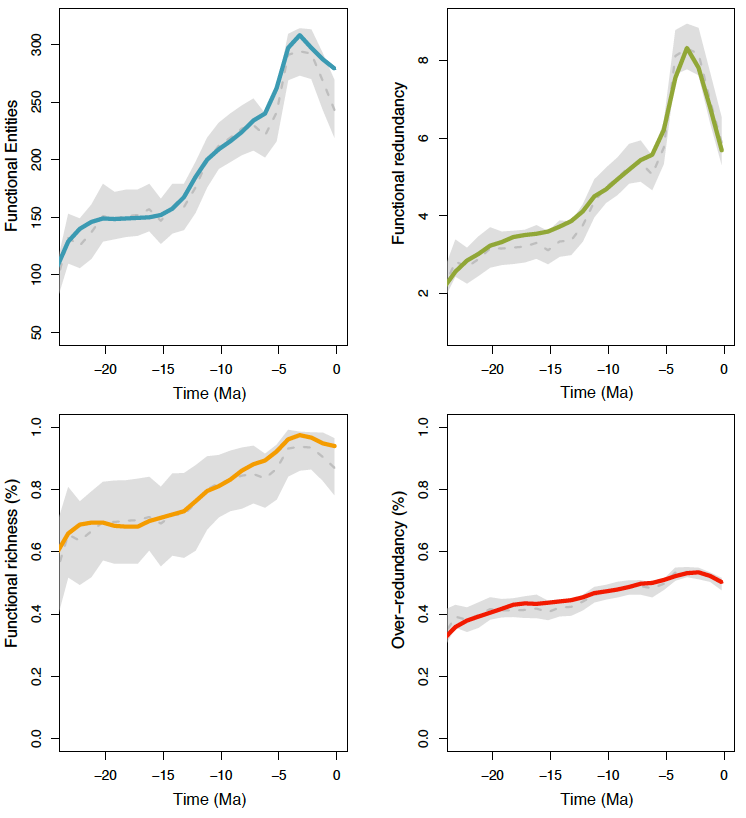


**Figure S3. Observed changes in functional diversity compared with random expectations.** Thick colored lines show values obtained empirically, grey polygons show values obtained by randomizing the identity of species, dashed grey line show mean, random values. Time is in million years.

**
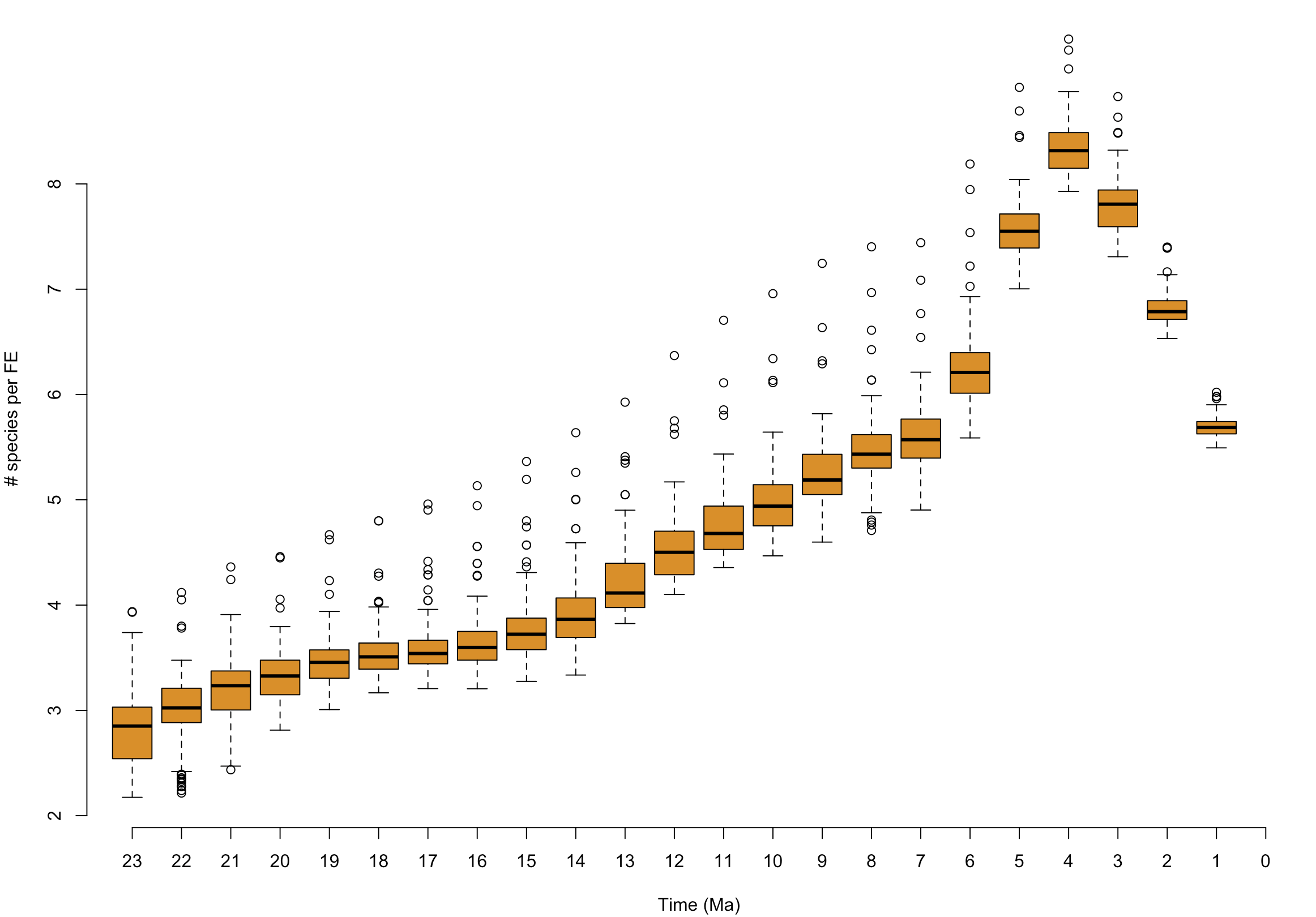
**

**Figure S4. Functional redundancy (FR) increases throughout the Miocene and Pliocene and decreases from the late Pliocene and throughout the Pleistocene.** FR is defined as the mean number of species per functional entity (FE). Time is in million years.

**
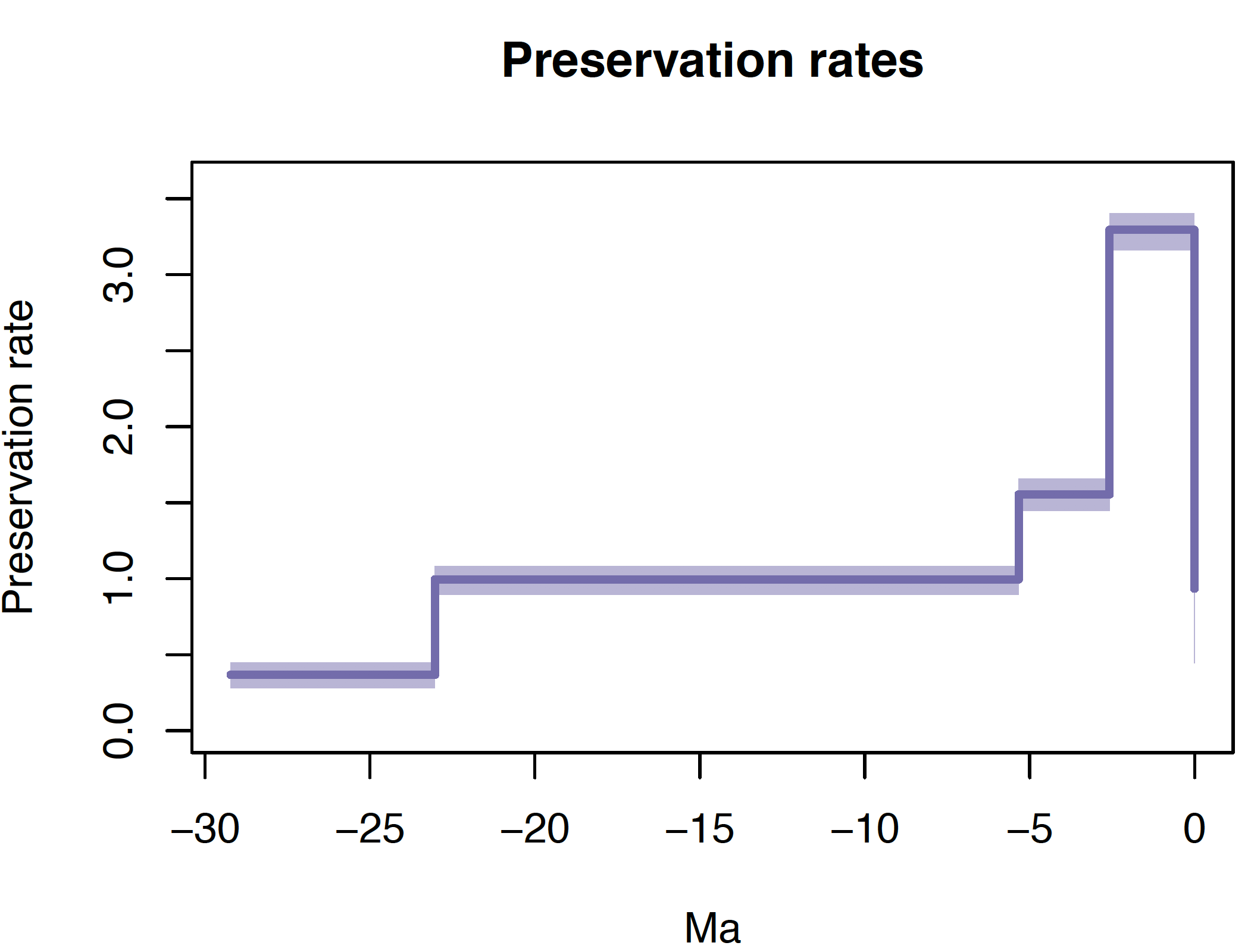
**

**Figure S5. Preservation rates through time as inferred by PyRate.** The posterior mean estimate of the preservation rate and 95% credible interval was inferred by epochs and quantifies the expected number of fossil occurrences per sampled lineage per million year. The preservation model also included rate variation across lineages. Time is in million years.
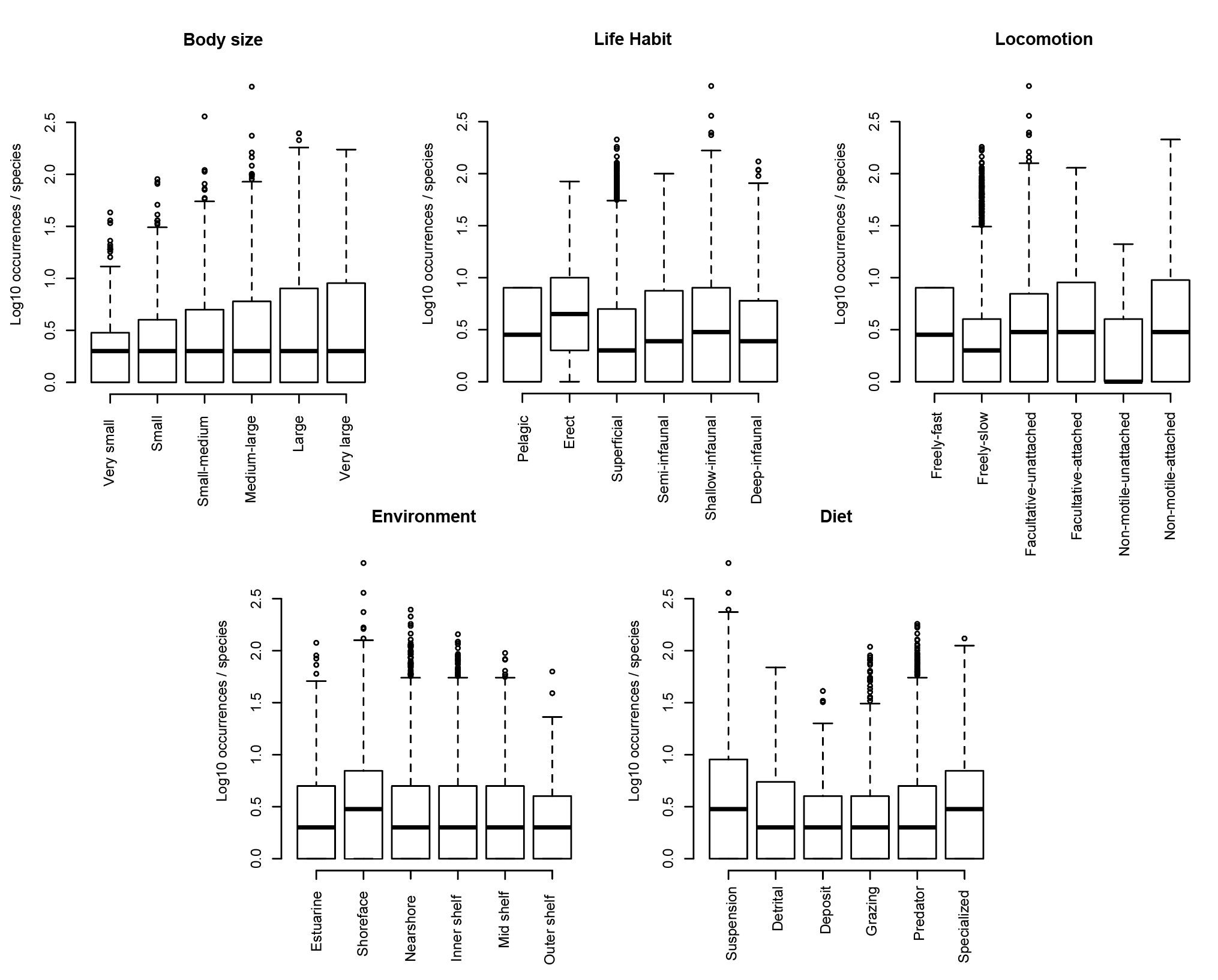
 **Figure S6. Occurrences per species (log-transformed) across trait states.** See table S1 for more details.

**
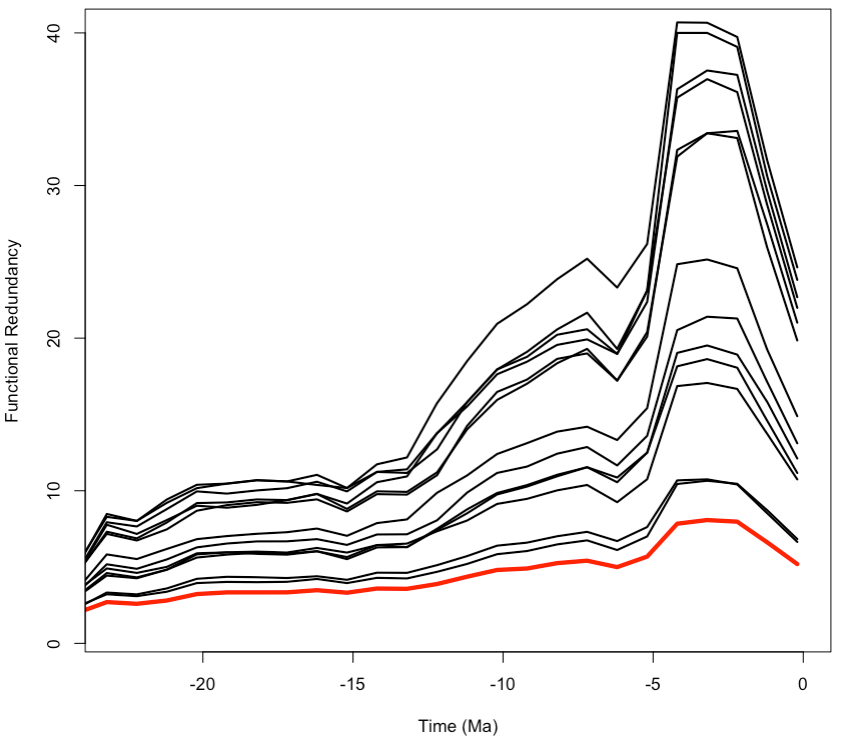
**

**Figure S7. Patterns of functional redundancy over time are maintained when altering the number of traits used.** Red line represents the observed value using all traits: body size, life habit, locomotion, environment and diet (also see figure S4). Black lines represent the values when one (e.g., diet) or two traits were dropped (e.g., diet and locomotion).

**
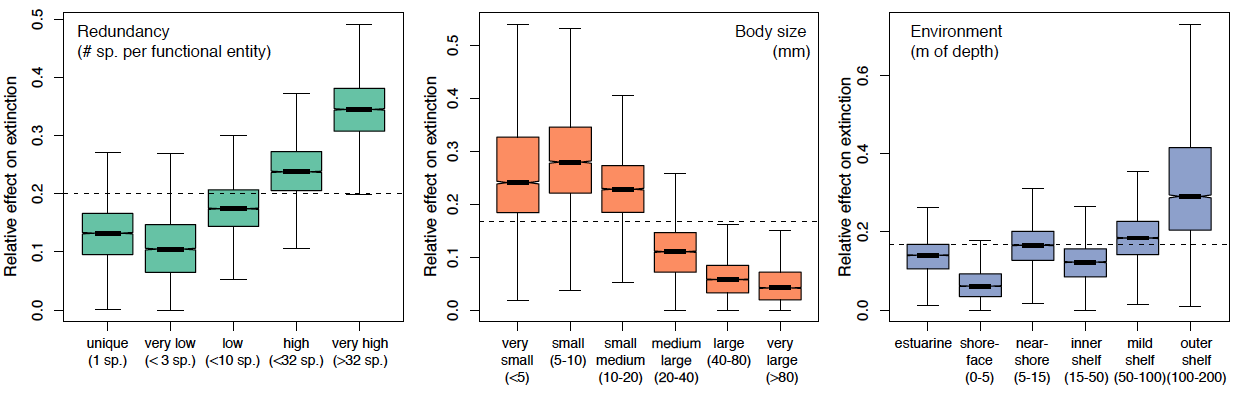
Figure S8.** Relative effect of redundancy, body size and environment on extinction rates, based on subsampled datasets, in which low, intermediate and high occupancy species were equally represented. As in figure 2 of the main text, the Y-axis corresponds to the Dirichlet-distributed multipliers (supplementary methods; equation 1 in main text). Dashed line is the expected value of the multipliers under a null model where the trait has no effect on extinction. The effects of the traits obtained from subsampled data are consistent with those inferred from the full dataset (figure 2).

**Table S1. Traits assigned to Caribbean mollusks. All traits are ordered factors.**

| **Function** | **Trait** | **Modality/Unit** |
| --- | --- | --- |
| Biology | Body size | 1. Very small (0-5 mm) 2. Small (5-10 mm) 3. Small-medium (10-20 mm) 4. Medium-large (20-40 mm)  5. Large (40-80 mm)  6. Very large (80-700 mm) |
| Habitat | Life habit | 1. Pelagic 2. Erect 3. Superficial  4. Semi-infaunal  5. Shallow-infaunal  6. Deep-infaunal |
|  | Locomotion | 1. Freely-fast  2. Freely-slow  3. Facultative-unattached  4. Facultative-attached  5. Non-motile-unattached  6. Non-motile-attached |
|  | Environment | 1. Estuarine (lower salinity/marginal marine environments)  2. Shoreface (0-5 m; rocky intertidal and beach settings)  3. Nearshore (5-15 m; shallow subtidal or transition zones)  4. Inner shelf (15-50 m)  5. Mid shelf (50-100 m)  6. Outer shelf (100-200 m) |
| Feeding | Diet | 1. Suspension  2. Detrital  3. Deposit  4. Grazing  5. Predator  6. Specialized (chemo or photo-symbiotic) |

**Table S2. Frequency of observed > null expectation for functional diversity indices computed across 1,000 null replicates.** Bold denote statistical significance (P < 0.025 in red indicating instances of observed values significantly lower than expected; P > 0.975 in blue indicating observed values significantly higher than expected).

| **Time (Ma)** | **Functional Entities** | **Functional redundancy** | **Functional richness** | **Over-**  **redundancy** |
| --- | --- | --- | --- | --- |
| 23 | 0.268 | **0.02** | 0.863 | 0.215 |
| 22 | 0.984 | 0.871 | 0.731 | **0.005** |
| 21 | 0.906 | 0.842 | 0.471 | 0.33 |
| 20 | 0.382 | 0.686 | 0.37 | 0.334 |
| 19 | 0.459 | 0.885 | 0.32 | 0.243 |
| 18 | 0.372 | 0.962 | 0.292 | 0.601 |
| 17 | 0.371 | 0.981 | 0.376 | 0.852 |
| 16 | 0.132 | 0.95 | 0.666 | 0.89 |
| 15 | 0.735 | 0.999 | 0.52 | 0.764 |
| 14 | 0.462 | 0.995 | 0.577 | 0.964 |
| 13 | 0.889 | 1 | 0.568 | 0.889 |
| 12 | 0.893 | 0.994 | 0.451 | 0.93 |
| 11 | 0.55 | 0.792 | 0.36 | 0.826 |
| 10 | 0.326 | 0.337 | 0.493 | 0.652 |
| 9 | 0.317 | 0.396 | 0.706 | 0.434 |
| 8 | 0.289 | 0.354 | 0.844 | 0.442 |
| 7 | 0.68 | 0.654 | **0.969** | 0.492 |
| 6 | **0.998** | **1** | **0.983** | 0.832 |
| 5 | **1** | **0.99** | 0.924 | 0.974 |
| 4 | 0.816 | **0** | **0.978** | 0.915 |
| 3 | **0.99** | 0.543 | 0.958 | **0.022** |
| 2 | 0.763 | **0.011** | **0.977** | 0.233 |
| 1 | **0.996** | 0.17 | **0.996** | 0.515 |
| 0 | **1** | 0.103 | 0.863 | 0.739 |

**Table S3. Absolute mean values of functional entities (FEs) and functional redundancy (FR: species per FE).**

| **Time (Ma)** | **FEs** | **FR** |
| --- | --- | --- |
| 23 | 128.33 | 2.59 |
| 22 | 137.94 | 2.81 |
| 21 | 144.22 | 2.98 |
| 20 | 147.27 | 3.19 |
| 19 | 147.35 | 3.34 |
| 18 | 149.8 | 3.48 |
| 17 | 151.54 | 3.56 |
| 16 | 154.03 | 3.61 |
| 15 | 156.85 | 3.67 |
| 14 | 162.95 | 3.8 |
| 13 | 171.97 | 3.95 |
| 12 | 187.79 | 4.25 |
| 11 | 202.3 | 4.56 |
| 10 | 211.04 | 4.79 |
| 9 | 219.37 | 5.02 |
| 8 | 227.98 | 5.25 |
| 7 | 235.45 | 5.47 |
| 6 | 242.4 | 5.61 |
| 5 | 264.14 | 6.29 |
| 4 | 296.71 | 7.59 |
| 3 | 308.05 | 8.34 |
| 2 | 298.73 | 7.8 |
| 1 | 287.86 | 6.82 |
| 0 | 279.05 | 5.7 |
| Mode | 279.05 | 5.7 |
| Mean | 204.71 | 4.77 |

Supplementary references

[69] Zhu, L., Fu, B., Zhu, H., Wang, C., Jiao, L. & Zhou, J. 2017 Trait choice profoundly affected the ecological connclusions drawn from the funnctional diversity measures. *Scientific Reports* **7**, e3643.

[70] Lefcheck, J.S., Bastazini, V.A.G. & Griffin, J.N. 2015 Choosing and using multiple traits in functional diversity research. *Environmental Conservationn* **42**, 104-107.

[71] Gelman, A., Bois, F. & Jiang, J. 1996 Physiological pharmacokinetic analysis using population modeling and informative prior distributions. *Journal of the American Statistical Association* **91**, 1400-1412.

Additional dataset (separate file)

Caribbean mollusk metadata including source, collection and occurrence IDs, taxonomic names, geographic locations of fossil occurrence, ages, formation information, and character states for five traits.
